# Development and validation of a predictive model for diagnosing EBER-positive lymphoma-associated hemophagocytic lymphohistiocytosis

**DOI:** 10.1186/s12885-025-13788-8

**Published:** 2025-03-05

**Authors:** Yuhong Yin, Wenzhi Zhang, Lizhen Zhao, Ying Li, Minchun Huang, Yu Han, Xiaoyan Wu

**Affiliations:** Department of Pediatrics, Union Hospital, Tongji Medical College, Huazhong University of Science and Technology, Wuhan, 430022 People’s Republic of China

**Keywords:** Epstein-Barr virus, Lymphoma, Hemophagocytic lymphohistiocytosis, Predictive model

## Abstract

**Purpose:**

This study aims to identify distinguishing factors between EBER-positive lymphoma-associated hemophagocytic lymphohistiocytosis and non-neoplastic EBV-associated hemophagocytic lymphohistiocytosis. Additionally, we developed and validated a predictive diagnostic model based on these factors.

**Methods:**

To evaluate the early identification of individuals with EBER-positive lymphoma-associated hemophagocytic lymphohistiocytosis versus non-neoplastic EBV-associated hemophagocytic lymphohistiocytosis, we carried out a retrospective cohort research. The medical records system included 148 individuals’ diagnoses of EBV-associated hemophagocytic lymphohistiocytosis between 2015 and 2023.

**Results:**

In this study, 148 patients were included, 75 of whom had non-neoplastic EBV-associated hemophagocytic lymphohistiocytosis and the remaining 73 had EBER-positive lymphoma-associated hemophagocytic lymphohistiocytosis. The highest AUC, with a good predictive value, was found for IL-10 > 39.87 pg/ml in separating EBER-positive lymphoma-associated hemophagocytic lymphohistiocytosis from non-neoplastic EBV-associated hemophagocytic lymphohistiocytosis. The diagnosis of EBER-positive lymphoma-associated hemophagocytic lymphohistiocytosis was influenced by platelets < 33.5*10^9^/L, IL-6 > 20.79 pg/ml, and IFN-γ > 12.12 pg/ml as independent variables. These factors were combined with the predictive value of IL-10 > 39.87 pg/ml to establish the predictive model of the nomogram for diagnosis. The training set’s and validation set’s areas under the ROC curves were 0.825 and 0.812, respectively, showing that the model had good discrimination, a well-calibrated model, and a clinically valid model as indicated by the clinical decision curve.

**Conclusion:**

The results of this study showed that the prediction model based on platelets < 33.5*10^9^/L, IL-6 > 20.79 pg/ml, IFN-γ > 12.12 pg/ml, and IL-10 > 39.87 pg/ml could more accurately distinguish between EBER-positive lymphoma-associated hemophagocytic lymphohistiocytosis and non-neoplastic EBV-associated hemophagocytic lymphohistiocytosis. This could aid clinicians in the early detection and convenient individualization of treatment for EBV-associated hemophagocytic lymphohistiocytosis.

## Introduction

The virus, known as Epstein-Barr virus (EBV), is a double-stranded DNA virus that attaches itself to the CD21 receptor on B-lymphocytes through the glycoprotein gp350 in the viral envelope. It initially infects B-lymphocytes, although it can also infect T- or NK-cells [1]. EBV infection commonly presents as infectious mononucleosis and asymptomatic infections in organisms, however it can also present as hemophagocytic lymphohistiocytosis (HLH) or EBV-associated lymphoproliferative illness in a small percentage of organisms. The development and clinical management of these disorders are complicated by the interaction between the immunological status of the host and the biological characteristics of cells infected with EBV [2].

HLH is an overreacting condition brought on by aberrant lymphocyte, macrophage, and monocyte activation, proliferation, and production of high amounts of inflammatory cytokines as a result of an inherited or acquired aberration of immunoregulatory function [3]. Primary and secondary categories are applicable. Malignant tumors, autoimmune illnesses, infections, and other conditions can all lead to secondary hemophagocytic lymphohistiocytosis (sHLH) [4]. Any patient with HLH may have a tumor as a trigger; lymphoma is the most prevalent type, accounting for about 56% of instances of malignancy-associated HLH [5]. The diagnosis of other causes of HLH, especially infection-associated HLH, cannot rule out the presence of malignancy-associated HLH. EBV is the most frequent cause of infection-associated HLH and is also frequently observed in malignancy-associated HLH (such as EBV-driven lymphomas) [6].

Due to the overlapping clinical symptoms and laboratory test results between EBER-positive lymphoma-associated hemophagocytic lymphohistiocytosis (EBER-positive LAHS) and non-neoplastic EBV-associated hemophagocytic lymphohistiocytosis (non-neoplastic EBV-HLH), EBER-positive lymphomas are frequently misdiagnosed [7]. According to earlier research, HLH occurred either before or simultaneously with EBER-positive lymphoma in 84.3% of patients with EBER-positive LAHS [8]. Consequently, screening for an underlying lymphoma history is necessary in individuals with sHLH, even in cases when an EBV infection has been identified. PET/CT and pathological biopsies are required to confirm the existence of lymphoma in individuals presenting with EBV-associated hemophagocytic lymphohistiocytosis. Patients with lymphoma with HLH typically have acute disease start, serious state, rapid progression, prolonged or even problematic diagnosis, and poor response to standard HLH therapies [9–11]. The development of a diagnostic predictive model for EBER-positive LAHS is required because of its relatively uncommon onset and the paucity of information that distinguishes it from non-neoplastic EBV-HLH. In this work, we improved the understanding of clinicians regarding the early identification, risk stratification, and therapeutic options of EBER-positive LAHS by developing a diagnostic prediction model to differentiate it from non-neoplastic EBV-HLH using laboratory data from patients with EBV-associated hemophagocytic lymphohistiocytosis.

## Methods

### Study population

Retrospective data collection was conducted on 154 patients with EBV-associated hemophagocytic lymphohistiocytosis who were treated at Union Hospital of Tongji Medical College, Huazhong University of Science and Technology, China, between 2015 and 2023. The following criteria were used to determine inclusion: non-neoplastic EBV-HLH: age range of 1 to 80 years; meeting five or more of the eight diagnostic criteria for HLH: (1) Fever; (2) Cytopenia (reduction of at least two cell lines in peripheral blood), defined as hemoglobin < 90 g/L, platelet count < 100 × 10^9^/L, and neutrophils < 1.0 × 10^9^/L; (3) Hypertriglyceridemia and/or hypofibrinogenemia (fasting triglycerides ≥ 3.0 mmol/L, fibrinogen ≤ 1.5 g/L); (4) Splenomegaly; (5) Hemophagocytosis in bone marrow, spleen, or lymph nodes without evidence of malignancy; (6) Reduced or absent NK cell activity; (7) Serum ferritin ≥ 500 µg/L; (8) Soluble CD25 ≥ 2.4 × 10^6^ U/L [12]; there must be evidence of EBV infection to meet at least one of the following two requirements: (1) serological antibody testing suggestive of primary acute EBV infection (see Diagnostic Evidence of IM); and (2) molecular biology techniques such as PCR, in situ hybridization, and Southern hybridization from the patient’s serum, bone marrow, lymph nodes, and other affected tissues are positive for EBV, such as serum or plasma EBV-DNA positivity and positive immunohistochemical staining for EBV-EBERs in situ or EBV-LMP1 in the affected tissues. EBER-positive LAHS is defined as follows: patient age range of 1 to 80 years; lymphoma histological diagnosis; EBV positivity determined by EBER in situ hybridization; and meeting five or more of the eight HLH diagnostic criteria. Two main exclusion criteria were patients without baseline EBV data. And primary HLH due to genetic disorders. All participants in this study provided informed consent. The study was reviewed and approved by the Medical Ethics Committee and was performed in accordance with the Declaration of Helsinki (2021.02.22, UHCT-IEC-SOP-016-03-01).

### Study grouping

**Fig. 1:**
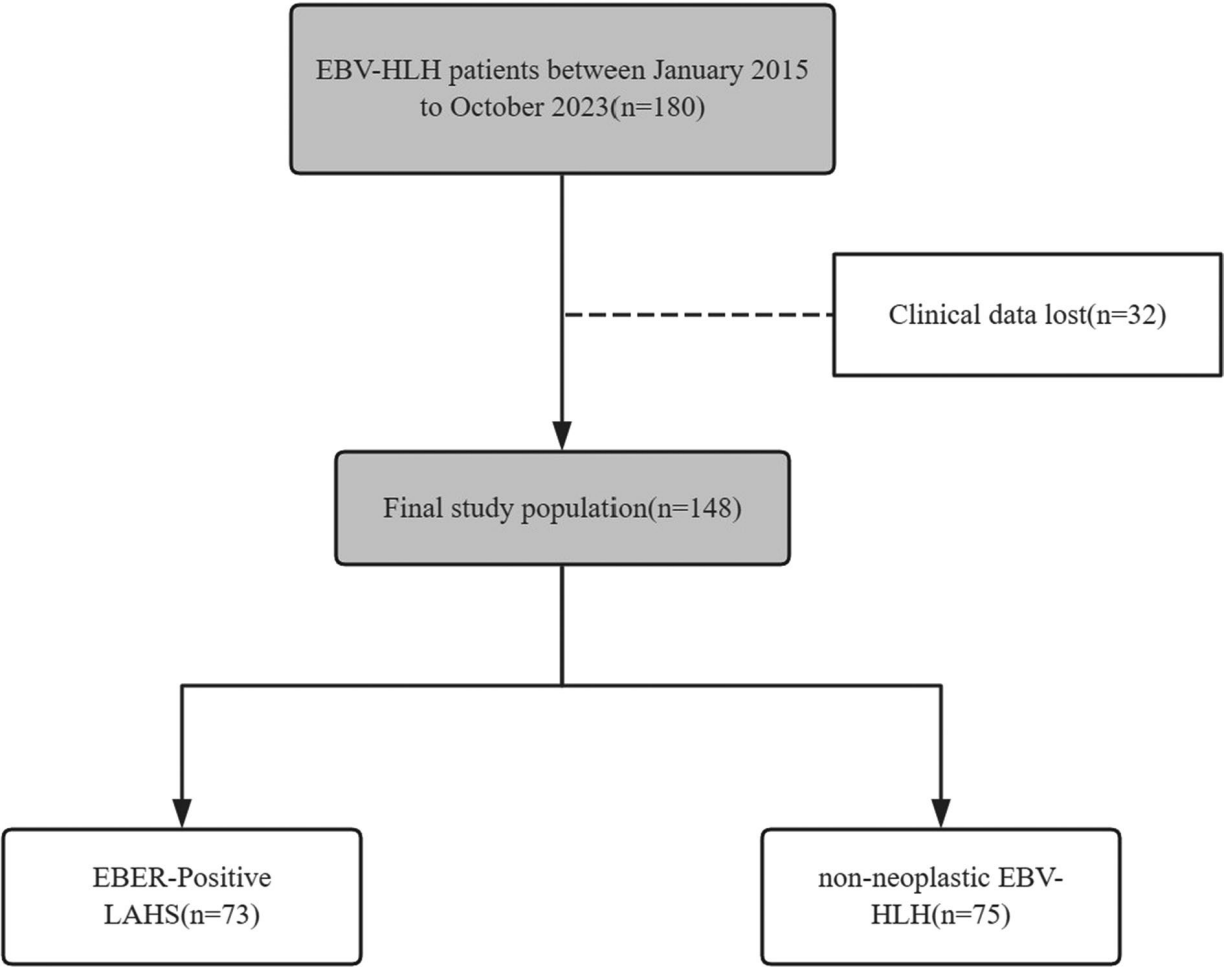
Data screening flowchart

Participants in the study were grouped according to their histological diagnosis of lymphoma: those who satisfied this criterion were assigned to the EBER-positive LAHS group, while those who did not fit the above parameters were assigned to the non-neoplastic EBV-HLH group. (Fig. 1)

### Data collection

From the patient’s medical records in the electronic medical record system of the Union Hospital of Tongji Medical College, Huazhong University of Science and Technology, China, the most recent laboratory values assessed before treatment for HLH or lymphoma were retrieved. The first number that was recorded within 72 h of the start of treatment was used in cases where no data were available.

### Statistical analyses

Continuous variables’ normality is examined using the D’Agostino-Pearson test. The Student t-test is used if the value passes both the equal variance and normality tests; if not, the Mann-Whitney test is employed. The chi-square test was used to compare categorical variables. In order to assess and externally validate the models, survival curves were produced using the Kaplan-Meier method and the log-rank test; diagnostic prediction models were created using LASSO regression analysis and multifactorial analysis; and Receiver operating characteristic (ROC) curves, calibration curves, and clinical decision curve analysis (DCA) were plotted using the data from the training set and validation set. R (4.3.2) and RStudio, SPSS software (R26.0.0.0), and Graphpad Prism (9.5.0) were used for all statistical computations. When *P* < 0.05, all statistical analysis results were deemed statistically significant.

## Results

### Baseline characteristics

**Fig. 2:**
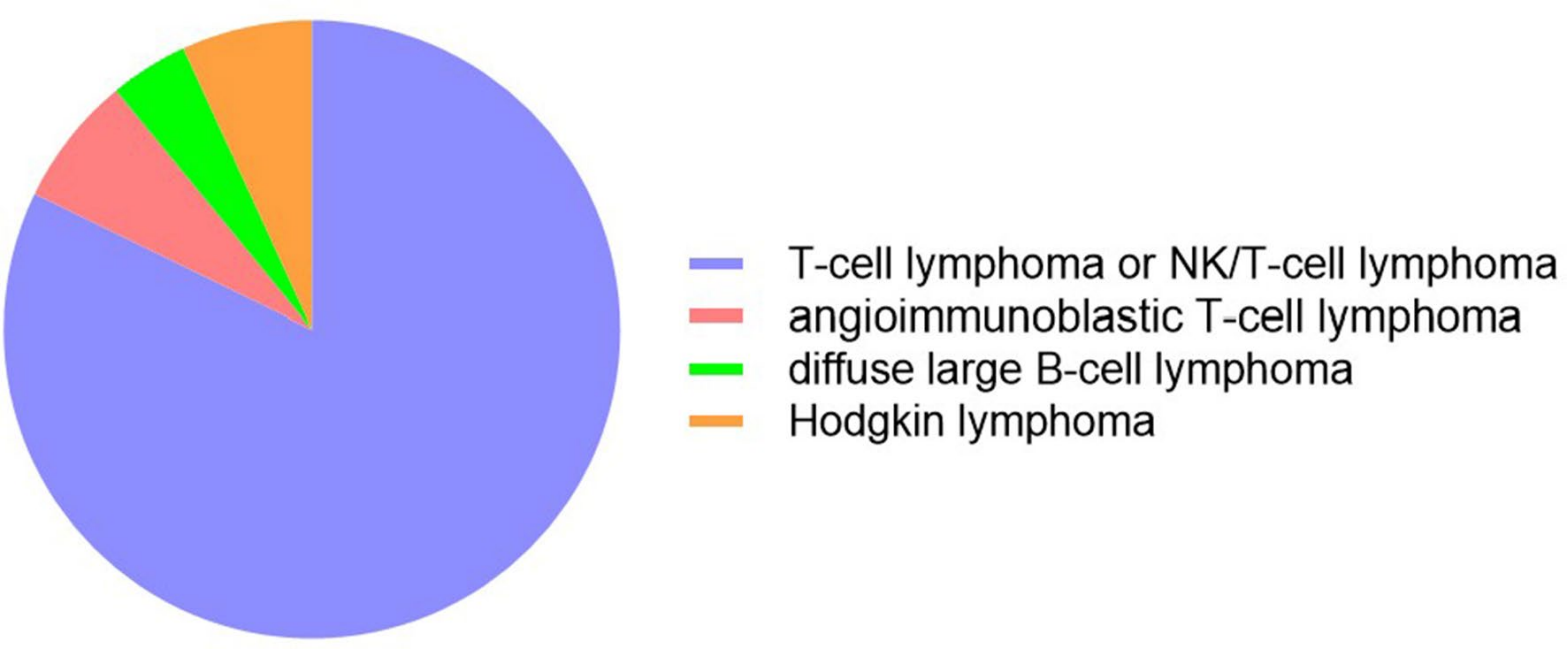
Lymphoma pathological subtypes in EBER-positive LAHS patients

The following findings were obtained from a statistical analysis of the 148 patients with EBV-associated hemophagocytic lymphohistiocytosis that were included: 38 (26%) of the patients were under the age of 18, 110 (74%) were over the age of 18, 96 (64.86%) were male, and 52 (35.14%) were female. In the non-neoplastic EBV-HLH group, there were 75 cases; 24 (32%) of these patients were under the age of 18, 51 (68%) were over the age of 18, 48 were male, and 27 were female. In the EBER-positive LAHS group, there were 73 cases; 14 (19%) of these patients were under the age of 18, 59 (81%) were patients over the age of 18, 48 cases in males, and 25 cases in females. Among them, 60 cases were T-cell lymphoma or NK/T-cell lymphoma (82.4%), 5 cases were angioimmunoblastic T-cell lymphoma (6.8%), 3 cases were diffuse large B-cell lymphoma (4%), and 5 cases were Hodgkin lymphoma (6.8%) (Fig. 2). The baseline characteristics were compared between the EBER-positive LAHS group and the non-neoplastic EBV-HLH group (Table 1). When HLH was first diagnosed, the following parameters were found to be statistically different between the two groups: c-reactive protein (CRP), platelet count, serum total bilirubin (STB), albumin, triglycerides (TG), lactate dehydrogenase (LDH), blood urea nitrogen (BUN), blood potassium, ferritin, IL-6, IL-10, IFN-γ, and plasma EBV-DNA load (all *P* < 0.05). The remaining factors were found to have no statistically significant differences between the groups (*P* > 0.05).

**Table 1:** Differences in baseline characteristics between groups with EBV-associated hemophagocytic lymphohistiocytosis

	non-neoplastic EBV-HLH (*n* = 75)	EBER-positive LAHS (*n* = 73)	*p*
Sex, n (%)			0.959
Male	48 (64)	48 (66)	
Female	27 (36)	25 (34)	
Age, n (%)			0.11
≤ 18 years > 18years	24 (32) 51 (68)	14 (19) 59 (81)	
Fever	65 (87)	69 (95)	0.103
Lymphadenectasis	61 (81)	55 (75)	0.376
Hepatomegaly	32 (43)	40 (55)	0.140
Splenomegaly	67 (89)	61 (84)	0.304
Disturbance of consciousness	5 (7)	4 (5)	1
CRP	20 (7.24, 54.8)	32.7 (14.1, 63.5)	0.044
WBC	2.77 (1.64, 5.13)	2.52 (1.5, 4.44)	0.396
NE	1.45 (0.58, 2.77)	1.6 (0.85, 2.5)	0.395
MON	0.2 (0.08, 0.57)	0.17 (0.09, 0.31)	0.396
HB	94.84 ± 20.73	91.74 ± 21.59	0.375
PLT	70 (43, 126)	48 (26, 87)	0.001
STB	15.6 (9.95, 32.9)	24.9 (14, 52.3)	0.021
DBIL	7.8 (3.9, 19.75)	11.5 (5.9, 31.1)	0.103
ALT	73 (35.5, 208)	77 (41, 148)	0.989
AST	72 (39.5, 201.5)	124 (50, 257)	0.083
ALB	30.5 (27.05, 35.15)	28.6 (25.6, 31.4)	0.019
LG	25.2 (20.9, 29.95)	23 (19.8, 27.8)	0.073
TC	3.08 ± 0.95	3.31 ± 0.9	0.125
TG	2 (1.54, 2.75)	2.42 (1.83, 3.89)	0.013
LDH	618 (401, 1015.5)	767 (450, 1736)	0.044
BUN	4.9 (3.32, 6.52)	5.8 (4.7, 7.67)	< 0.001
Cr	54 (42.6, 67.6)	58.9 (45.1, 77.6)	0.201
Na	135.9 (132.45, 137.7)	135 (130.7, 137.5)	0.255
K	3.82 ± 0.49	3.99 ± 0.56	0.048
Ca	2.02 (1.93, 2.12)	2 (1.89, 2.07)	0.231
Mg	0.84 ± 0.12	0.83 ± 0.16	0.688
DDimer	2.42 (1.49, 4.9)	3.24 (1.89, 6.76)	0.088
FDP	10.2 (4, 17.45)	10.9 (5.5, 28.32)	0.328
PT	14 (13.1, 15.55)	14.5 (13.4, 15.9)	0.156
APTT	42.8 (37.75, 48.8)	43 (37.6, 51.7)	0.89
INR	1.15 (1.04, 1.31)	1.2 (1.09, 1.32)	0.111
FIB	2.15 (1.38, 3.38)	1.93 (1.29, 2.69)	0.248
TT	19.8 (18.4, 24.5)	19.5 (17.8, 22.7)	0.438
SF	4379.6 (1972.8, 12773)	10774.8 (3521, 40000)	0.006
IL-6	11.48 (6.4, 25.99)	28.19 (15.37, 66.6)	< 0.001
IL-10	27.83 (8.07, 126.62)	152.68 (44.15, 312.66)	< 0.001
IL-2	2.17 (1.56, 3.35)	2.02 (1.41, 3.52)	0.837
IL-4	2.24 (1.61, 2.91)	2.27 (1.74, 3.18)	0.623
TNF-α	2.42 (1.59, 4.56)	3.02 (2.12, 5.74)	0.267
IFN-γ	4.45 (2.69, 10.63)	16.31 (6.6, 100.81)	< 0.001
PBMC EBV DNA	160,000 (14700, 1480000)	418,000 (31600, 3730000)	0.153
Plasma EBV DNA	12,000 (764, 81200)	60,700 (15000, 689000)	< 0.001

The optimal cut-off value for the diagnosis of EBER-positive LAHS was determined by ROC curve analysis applied to the continuous variables that were statistically different between the two groups. The maximum Yoden’s index for CRP at the time of HLH diagnosis was determined to be 0.24, corresponding to a value of 11.75 mg/L. This value was used as the optimal cut-off value for dividing CRP at the time of HLH into the groups CRP > 11.75 mg/L and CRP ≤ 11.75 mg/L. Together with CRP, PLT, STB, ALB, TG, LDH, BUN, K, SF, IL-6, IL-10, IFN-γ, and plasma EBV-DNA were split into two groups, each with the ideal cut-off value (Table 2).

**Table 2:** Optimal cut-off values for continuous variables

	Optimal cut-off value	Sensitivity	Specificity	Yoden’s index	AUC
CRP	11.75	0.84	0.41	0.24	0.61
PLT	33.50	0.41	0.86	0.28	0.66
STB	17.75	0.67	0.54	0.21	0.60
ALB	31.75	0.78	0.47	0.25	0.62
TG	3.77	0.30	0.86	0.17	0.59
LDH	750.50	0.58	0.65	0.23	0.58
BUN	5.33	0.67	0.63	0.30	0.67
K	4.29	0.29	0.85	0.14	0.58
SF	3518.75	0.77	0.49	0.26	0.63
IL-6	20.79	0.64	0.71	0.35	0.70
IL-10	39.87	0.78	0.60	0.38	0.68
IFN-γ	12.12	0.63	0.77	0.40	0.73
Plasma EBV DNA	13550.00	0.77	0.52	0.29	0.68

### Screening of potential factors for diagnosis of EBER-positive LAHS

**Fig. 3:**
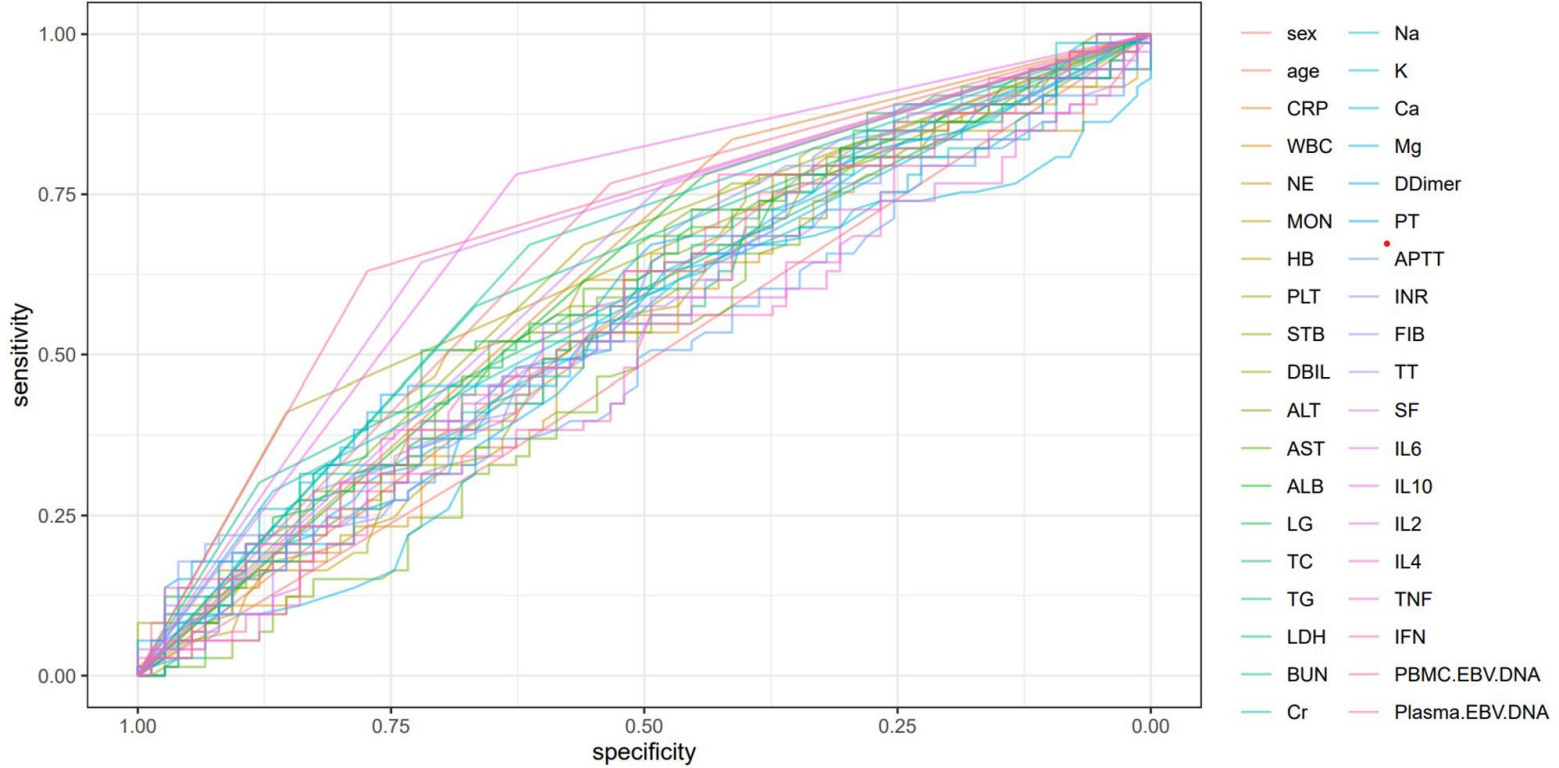
ROC curves for all included variables predicting diagnosis of EBER-positive LAHS

**Fig. 4:**
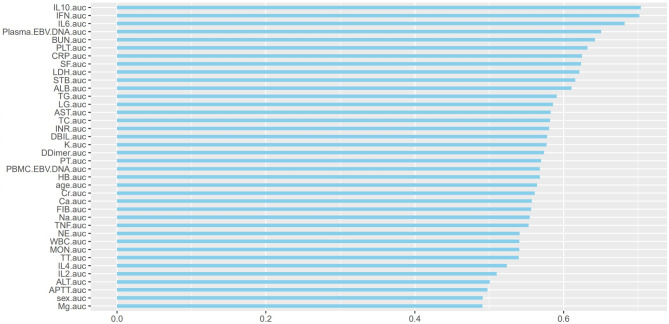
ROC curve area ranking for all variables

**Fig. 5:**
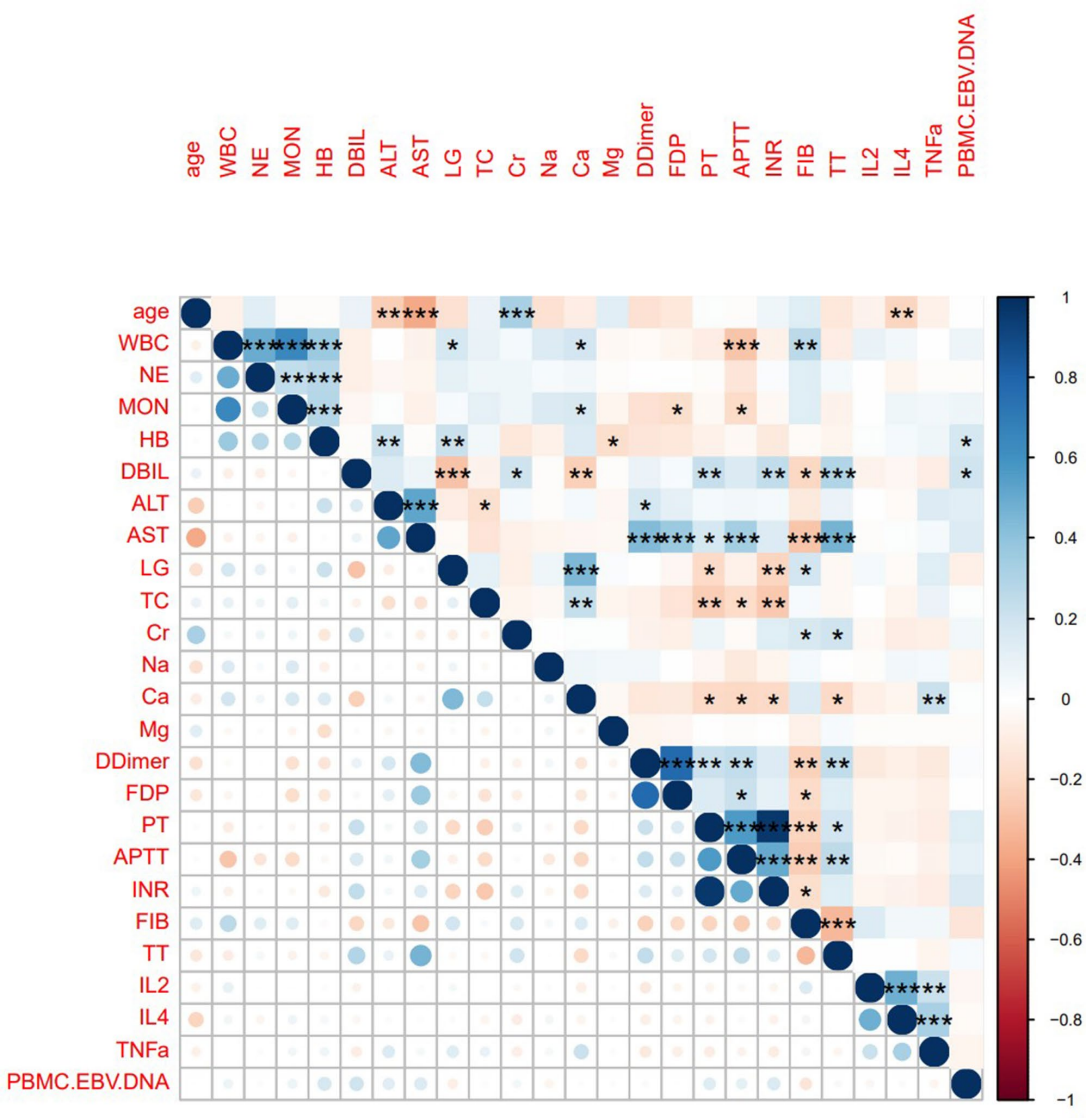
Correlation between continuous variables (the darker the colour, the stronger the correlation, the significance of correlation between variables is indicated by“*” means *P* < 0.05, “**” means *P* < 0.01, “****” means *P* < 0.001)

**Fig. 6:**
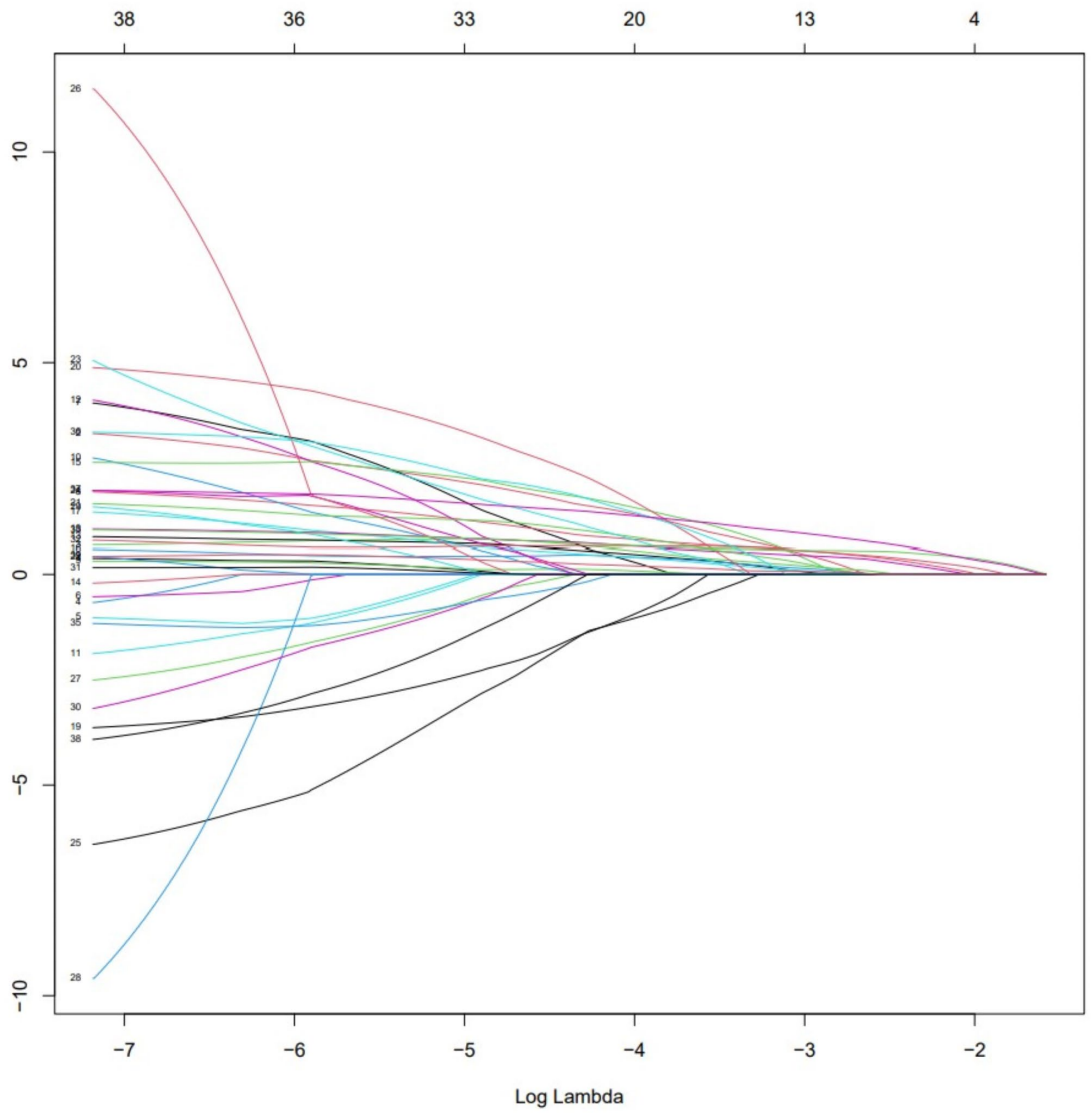
LASSO regression screening variable dynamic process diagram

**Fig. 7:**
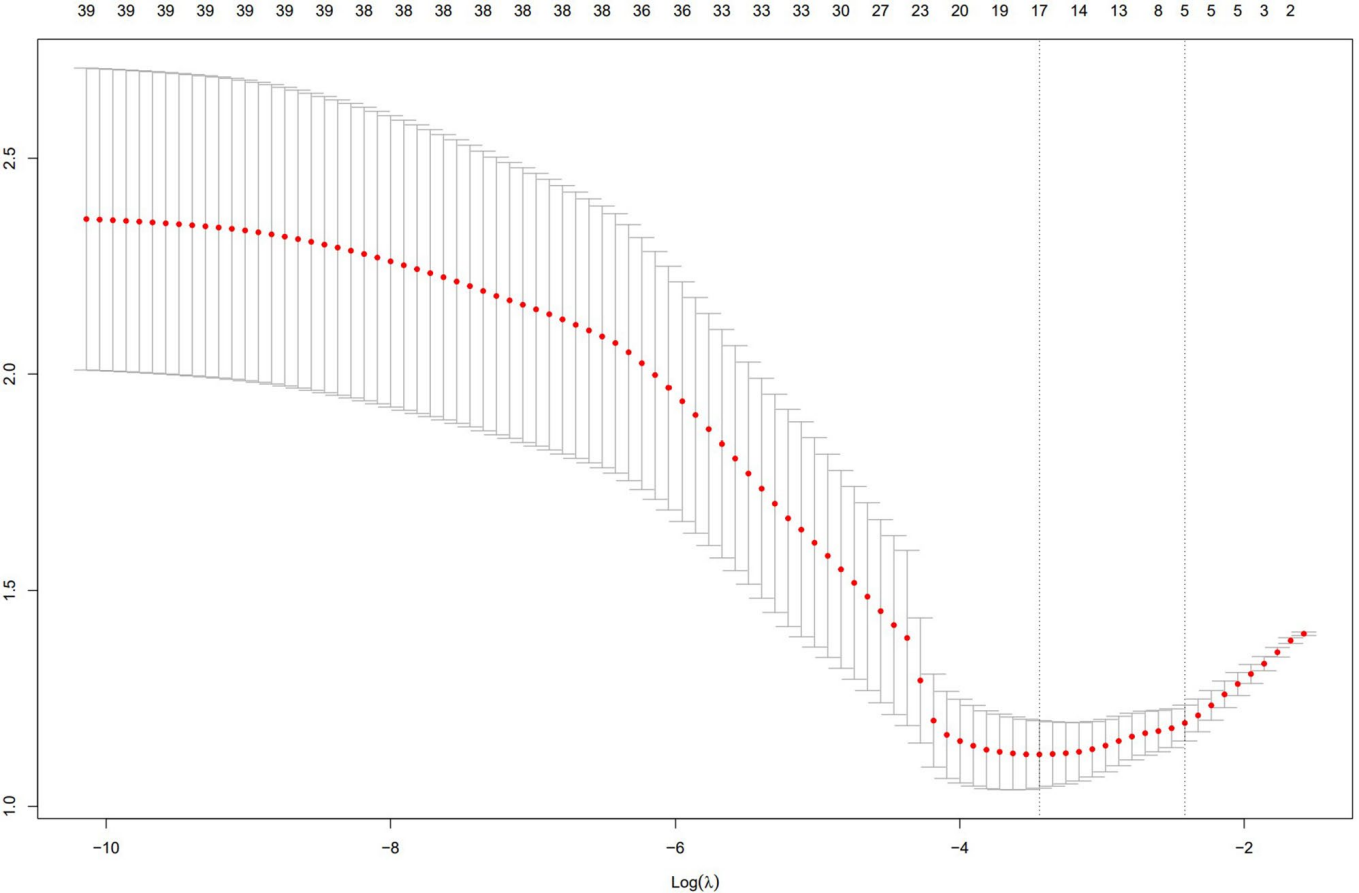
Cross-validation to select the best λ-process diagram

ROC curves and AUC values were continuous variables gathered in Tables 1 and 2, and all the variables were ranked in order to investigate the predictive power of each variable for a patient’s diagnosis of EBER-positive LAHS (Fig. 3). Of the single variables, the largest AUC of 0.703 was found for the prediction of a diagnosis of EBER-positive LAHS using IL-10 > 39.87 pg/ml (Fig. 4). Additionally, 25 continuous variables from the aforementioned variables were subjected to correlation tests, and relationships between a number of continuous variables were discovered (Fig. 5). Not all of these variables could be included in the logistic regression analysis to prevent overfitting. LASSO regression analysis was utilized to filter the variables and choose predictor variables. Cross-validation was employed to determine the ideal penalty coefficient (λ). With limited affecting factors and strong model performance, the five predictors with the greatest value of λ were platelets < 33.5*10^9^/L, urea nitrogen > 5.33 mmol/L, IL-6 > 20.79 pg/ml, IFN-γ > 12.12 pg/ml, and IL-10 > 39.87 pg/ml. In order to create a more meaningful prediction model for the diagnosis of EBER-positive LAHS, these five predictive criteria can be employed (Figs. 6 and 7).

Multifactorial logistic regression analysis was performed using the five predictors that were screened using LASSO regression. The findings indicated that PLT ≤ 33.5*10^9^/L, IL-6 > 20.79 pg/ml, and IFN-γ > 12.12 pg/ml were the independent influencing factors for the diagnosis of EBER-positive LAHS (Table 3).

**Table 3:** Multifactorial logistic regression analysis for the diagnosis of EBER-positive LAHS

	OR	S.E.	Wald Z	95% CI	*P*
PLT < 33.5*10^9^/L	1.25	0.62	2.00	0.93–1.70	0.045
BUN > 5.33mmol/L	1.04	0.57	1.82	0.87–1.25	0.069
IL-10 > 39.87pg/ml	0.99	0.53	1.88	0.85–1.16	0.060
IL-6 > 20.79pg/ml	1.41	0.52	2.71	1.13–1.74	0.007
IFN-γ > 12.12 pg/ml	1.45	0.59	2.45	1.17–1.79	0.015

### Development and validation of a diagnostic prediction model for EBER-positive LAHS

#### Training set and validation set baseline characteristics comparison

148 patient groups were randomly assigned using RStudio software for this study. Of them, 103 cases were allocated to the training set for the development of the diagnostic prediction model, while 45 cases were assigned to the validation set to assess the model’s performance in real-world applications. present the findings from the comparison of the variables in the two sets of data (Table 4). There was strong comparability between the two sets of data as none of the between-group differences in the variables between the two groups were statistically significant (*P* > 0.05).

**Table 4:** Training set and validation set baseline characteristics comparison

	Validation set (*n* = 45)	Training set (*n* = 103)	*P*
Diagnosis, n (%)			0.641
Non-neoplastic EBV-HLH	21 (47)	54 (52)	
EBER-positive LAHS	24 (53)	49 (48)	
Sex, n (%)			0.796
Male	28 (62)	68 (66)	
Female	17 (38)	35 (34)	
Age			0.666
≤ 18years > 18years	28 (27) 75 (73)	10 (22) 35 (78)	
Fever	41 (91)	93 (90)	1
Lymphadenectasis	39 (87)	77 (75)	0.105
Hepatomegaly	20 (44)	52 (50)	0.499
Splenomegaly	42 (93)	86 (83)	0.107
disturbance of consciousness	2 (4)	8 (8)	0.7
CRP, n (%)			0.311
CRP ≤ 11.75 mg/L	10 (22)	33 (32)	
CRP > 11.75 mg/L	35 (78)	70 (68)	
WBC	2.52 (1.63, 4.73)	2.85 (1.54, 4.81)	0.772
NE	1.69 (0.84, 3.18)	1.48 (0.62, 2.46)	0.348
MON	0.17 (0.11, 0.31)	0.19 (0.08, 0.48)	0.799
HB	94.11 ± 24.47	92.96 ± 19.63	0.781
PLT, n (%)			0.7
PLT > 33.5*10^9^/L	34 (76)	73 (71)	
PLT ≤ 33.5*10^9^/L	11 (24)	30 (29)	
STB, n (%)			0.838
STB ≤ 11.75umol/L	19 (42)	47 (46)	
STB > 11.75umol/L	26 (58)	56 (54)	
DBIL	11 (4.4, 23.9)	9.5 (4.7, 24.6)	0.823
ALT	101 (51, 166)	66 (34.5, 155.5)	0.11
AST	124 (48, 238)	88 (45, 226.5)	0.564
ALB, n (%)			1
ALB > 31.75 g/L	15 (33)	34 (33)	
ALB ≤ 31.75 g/L	30 (67)	69 (67)	
LG	26 (22.1, 30)	23.4 (20.4, 28.35)	0.099
TC	3.14 ± 0.9	3.24 ± 0.96	0.554
TG, n (%)			0.637
TG ≤ 3.77mmol/L	34 (76)	83 (81)	
TG > 3.77mmol/L	11 (24)	20 (19)	
LDH, n (%)			0.754
LDH ≤ 750.5U/L	26 (58)	55 (53)	
LDH > 750.5U/L	19 (42)	48 (47)	
BUN, n (%)			0.779
BUN ≤ 5.33mmol/L	20 (44)	50 (49)	
BUN > 5.33mmol/L	25 (56)	53 (51)	
Cr	55.4 (44.6, 65.9)	58.3 (43.2, 71.65)	0.75
Na	135 (130.1, 138)	135.2 (132.4, 137.55)	0.284
K, n (%)			0.974
K≤ 4.29mmol/L	35 (78)	82 (80)	
K > 4.29mmol/L	10 (22)	21 (20)	
Ca	1.96 (1.86, 2.08)	2.02 (1.94, 2.11)	0.095
Mg	0.83 (0.76, 0.91)	0.84 (0.74, 0.94)	0.802
DDimer	2.39 (1.54, 5.5)	3 (1.88, 6.23)	0.143
FDP	8.7 (4.5, 15.4)	12 (6.07, 26.9)	0.08
PT	14.1 (13.4, 16.3)	14.4 (13.2, 15.3)	0.712
APTT	42 (38.3, 48.5)	43.1 (37.05, 49.8)	0.92
INR	1.19 (1.06, 1.43)	1.17 (1.05, 1.29)	0.575
FIB	1.98 (1.29, 3.32)	1.99 (1.34, 3.12)	0.886
TT	19.8 (18, 24.2)	19.5 (18.1, 22.9)	0.602
SF, n (%)			0.547
SF ≤ 3518.75ug/L	14 (31)	39 (38)	
SF > 3518.75ug/L	31 (69)	64 (62)	
IL-6, n (%)			0.513
IL-6 ≤ 20.79pg/ml	22 (49)	58 (56)	
IL-6 > 20.79pg/ml	23 (51)	45 (44)	
IL-10, n (%)			0.55
IL-10 ≤ 39.87pg/ml	17 (38)	46 (45)	
IL-10 > 39.87pg/ml	28 (62)	57 (55)	
IL-2	2.02 (1.2, 3.26)	2.16 (1.44, 3.36)	0.371
IL-4	2.24 (1.78, 3.07)	2.27 (1.68, 3.12)	0.967
TNF-α	2.81 (1.59, 5.87)	2.87 (1.72, 4.5)	0.84
IFN-γ			0.813
IFN-γ ≤ 12.12pg/ml	27 (60)	58 (56)	
IFN-γ > 12.12pg/ml	18 (40)	45 (44)	
PBMC EBV DNA	340,000 (14200, 2820000)	267,000 (20700, 2155000)	0.879
Plasma EBV DNA, n (%)			0.668
Plasma EBV DNA ≤ 13550copies/ml	19 (42)	38 (37)	
Plasma EBV DNA > 13550copies/ml	26 (58)	65 (63)	

### Development of a predictive diagnostic model for EBER-positive LAHS

**Fig. 8:**
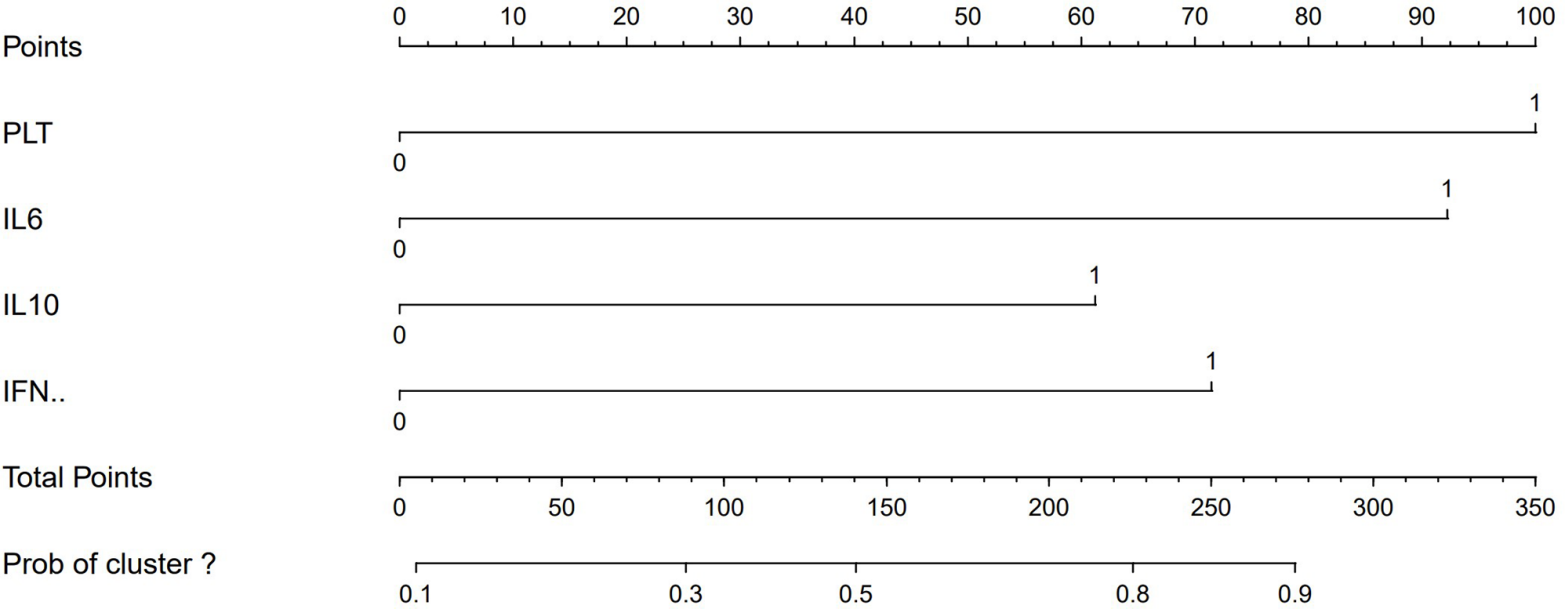
Diagnostic prediction of EBER-positive LAHS nomogram

Treatment options and timing are dependent on the early identification of patients diagnosed with non-neoplastic EBV-HLH and EBER-positive LAHS. Four parameters (PLT ≤ 33.5*10^9^/L, IL-6 > 20.79 pg/ml, IFN-γ > 12.12 pg/ml, and IL-10 > 39.87 pg/ml) were included in the diagnostic prediction model in this study, which was created based on the outcomes of the LASSO regression and multifactorial logistic regression analyses. This allowed for the creation of an easily navigable nomogram for clinicians (Fig. 8).

**Fig. 9:**
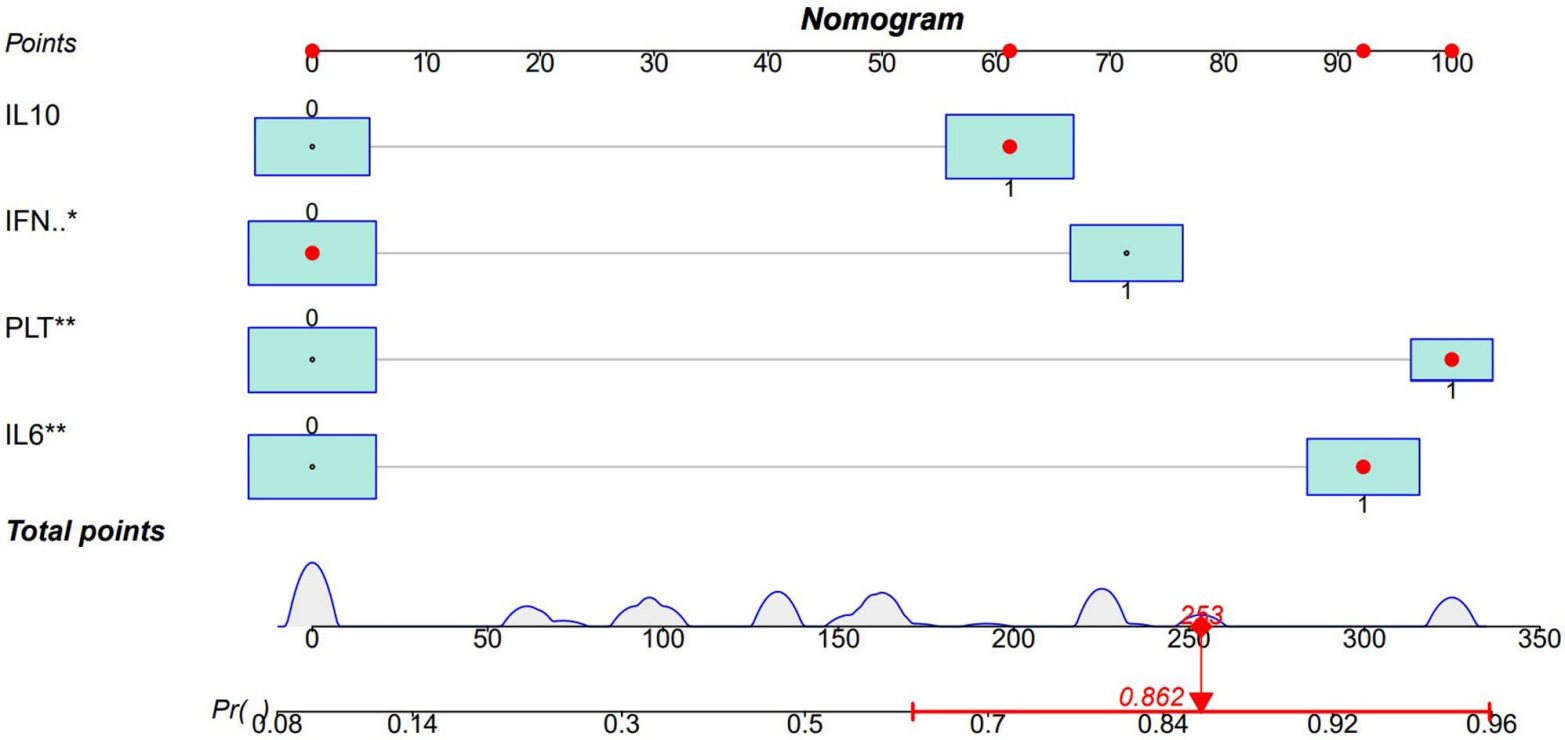
A case of EBER-positive LAHS. At the time of diagnosis, the patient had IL-10 levels > 39.87 pg/ml, IL-6 levels > 20.79 pg/ml, PLT < 33.5 × 10^9^/L, and IFN-γ levels < 12.12 pg/ml. Based on the nomogram, the patient’s total score was 253, predicting a probability of 0.862 for EBER-positive LAHS

**Fig. 10:**
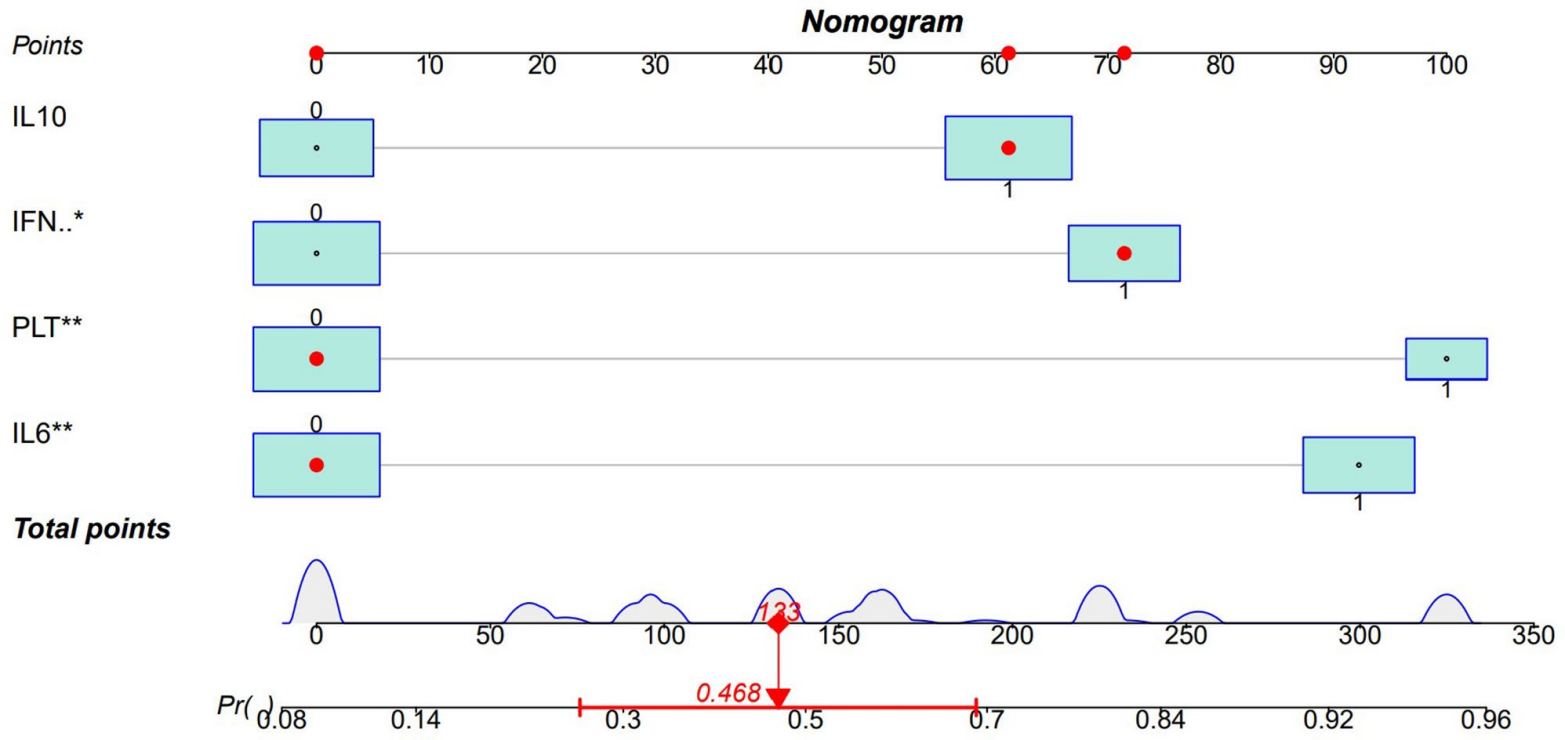
A case of non-neoplastic EBV-HLH. At the time of diagnosis, the patient had IL-10 levels > 39.87 pg/ml, IFN-γ levels > 12.12 pg/ml, IL-6 levels < 20.79 pg/ml, and PLT > 33.5 × 10^9^/L. According to the nomogram, the patient’s total score was 133, predicting a probability of 0.468 for EBER-positive LAHS

In this nomogram, the score for each parameter corresponds to a specific range of values. Physicians can use the patient’s specific test results to locate each parameter on the nomogram and calculate the corresponding score. By summing the scores of all parameters, the total score for the patient can be obtained. Based on the total score, physicians can assess the patient’s risk of developing EBER-positive LAHS. To better understand the application of this nomogram, Figs. 9 and 10 provide two examples of its use (Figs. 9 and 10).

### Evaluation and validation of a diagnostic prediction model for EBER-positive LAHS

**Fig. 11:**
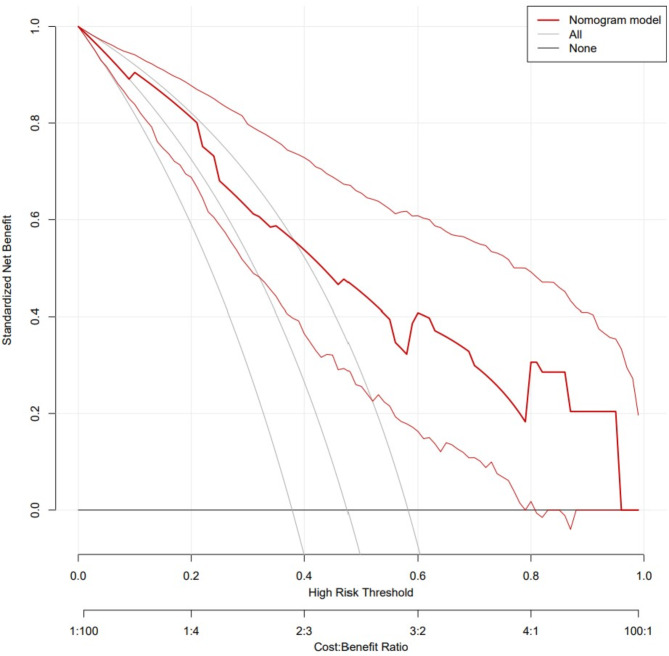
DCA curves for nomogram of training set

**Fig. 12:**
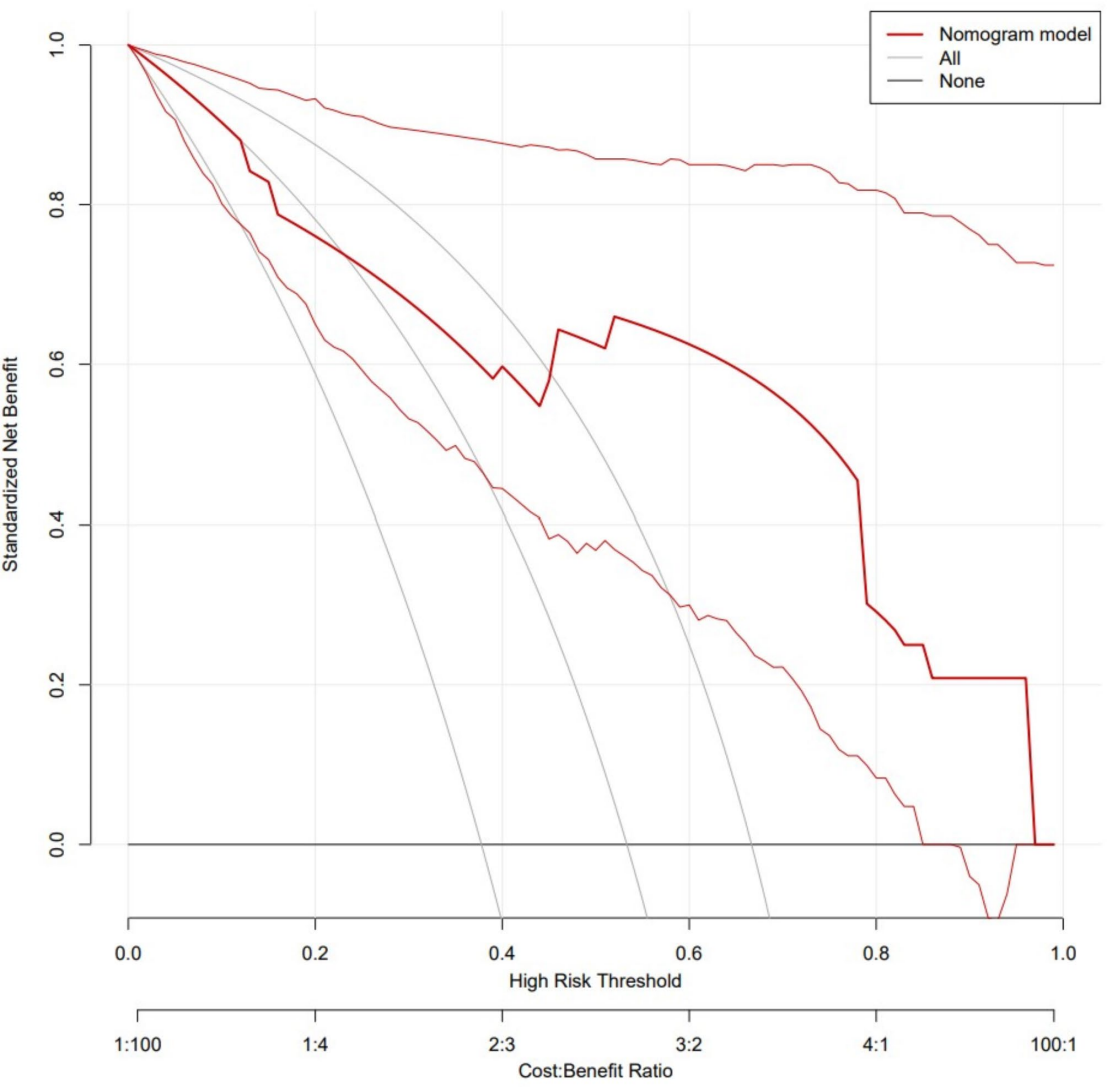
DCA curves for nomogram of validation set

**Fig. 13:**
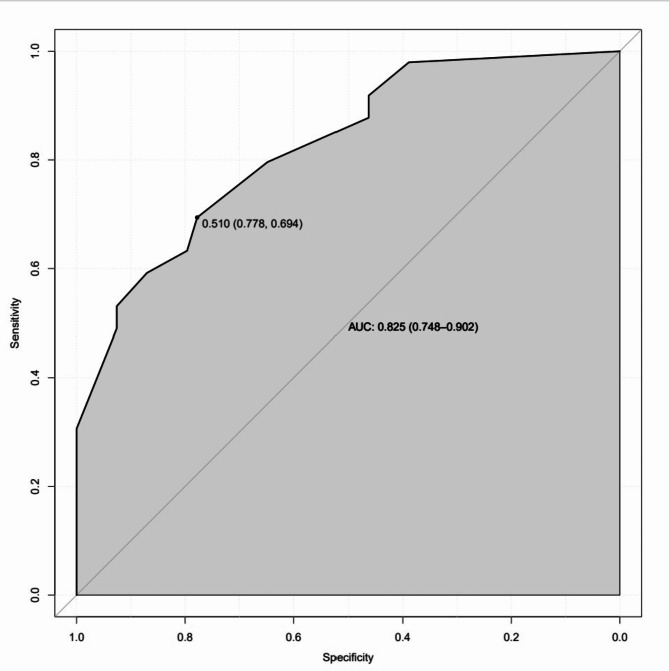
ROC curves for the training set

**Fig. 14:**
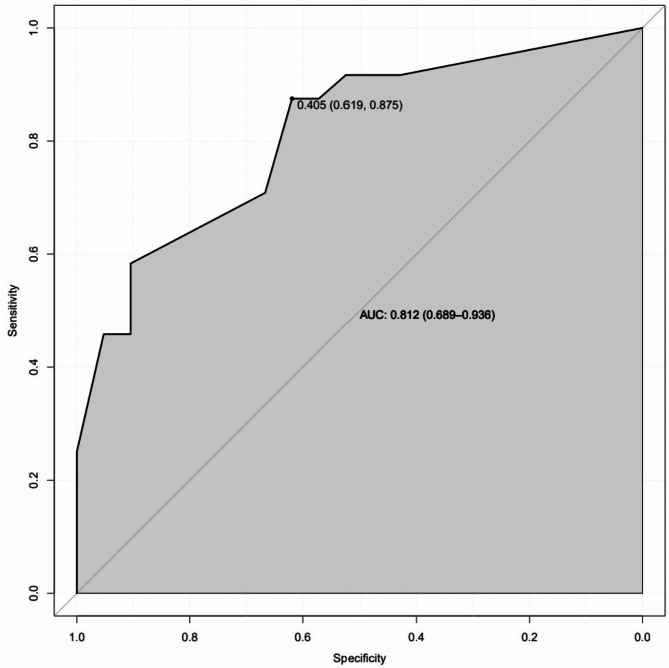
ROC curves for the validation set

**Fig. 15:**
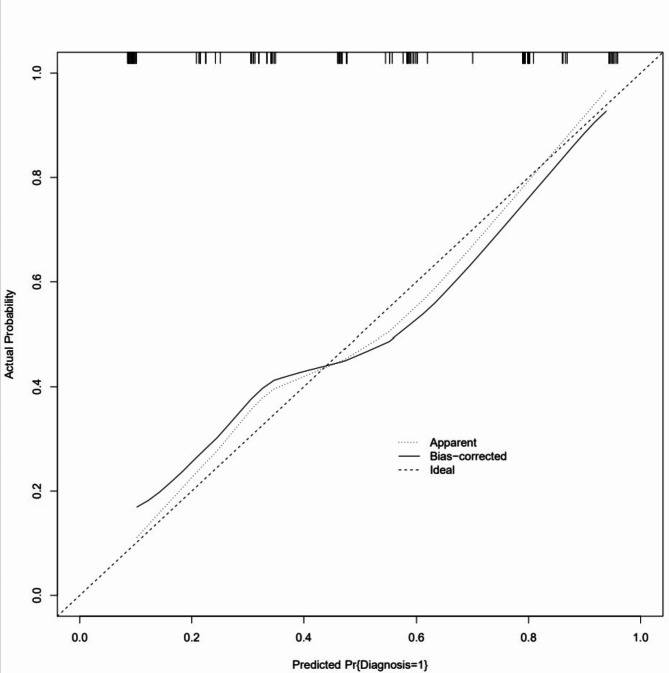
Training set goodness-of-fit test

**Fig. 16:**
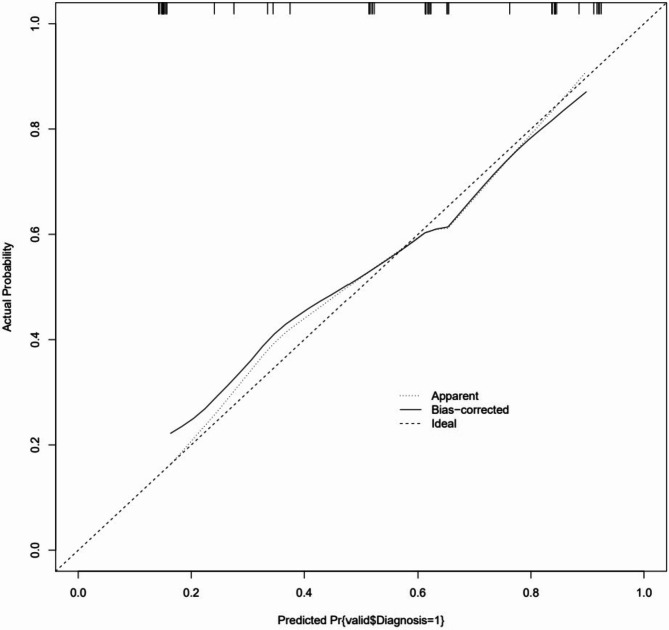
validation set goodness-of-fit test

ROC curves, calibration curves, and DCA curves were used to evaluate the clinical viability of the nomogram in order to further corroborate the validity of the diagnostic prediction model. In the interim, this study illustrated the model’s extrapolation by externally validating the diagnostic prediction model using the patient data from the validation set. The findings demonstrated the strong clinical validity of the diagnostic prediction model for EBER-positive LAHS created in this investigation (Figs. 11 and 12). With a sensitivity of 0.778 and a specificity of 0.694, the area under the ROC curve for the training set was 0.825 (0.748–0.902) (Fig. 13), and for the validation set, it was 0.812 (0.689–0.936) with a sensitivity of 0.619 and a specificity of 0.875 (Fig. 14). The model has good clinical validity, as evidenced by the calibration curves for the training and validation sets, which showed a high degree of agreement between the actual and projected probabilities. A high level of consistency suggests that the model has been calibrated correctly (Figs. 15 and 16).

## Discussion

The early mortality rate in patients with malignancy-associated HLH is significantly higher than in other types of HLH [13]. Therefore, early and accurate diagnosis, as well as scientific classification of HLH, is crucial [14]. Moreover, under the influence of EBV, some patients with malignancy-associated HLH initially present with EBV-associated hemophagocytic lymphohistiocytosis [7], especially those whose condition does not improve after receiving empirical HLH treatment. If the potential tumor etiology is not promptly identified [15], these patients often succumb to further deterioration of their condition [16]. Hence, it is of paramount importance to accurately distinguish between EBER-positive LAHS and non-neoplastic EBV-HLH. In this retrospective study, we found that a risk model based on platelet count < 33.5*10^9^/L, IL-6 > 20.79 pg/mL, IFN-γ > 12.12 pg/mL, and IL-10 > 39.87 pg/mL can effectively differentiate EBER-positive LAHS from non-neoplastic EBV-HLH. This model contributes to improving the accuracy of early diagnosis, guiding personalized treatment strategies, and enhancing clinical outcomes for patients.

Although the HLH-2004 diagnostic criteria are widely applied, they do not effectively and sensitively distinguish whether lymphoma is involved in the etiology of HLH. To address this issue, Tamamyan et al. proposed an expanded diagnostic criterion that includes new variables such as LDH, BUN, and ALB to improve diagnostic sensitivity [17]. This study also supports this viewpoint, particularly in patients with EBER-positive LAHS, where indicators such as CRP, STB, ALB, TG, BUN, LDH, and blood potassium were significantly elevated. This may be related to factors such as high tumor burden and concurrent infections [18–21]. Ferritin is one of the important biomarkers for HLH. Adi Zoref-Lorenz et al. established an index consisting of soluble CD25 > 3900 U/mL and ferritin > 1000 ng/mL, which can accurately identify malignancy-associatedpet HLH and highly predict mortality [22]. Our study also found that ferritin levels were significantly elevated in patients with EBER-positive LAHS. Previous studies have shown that in patients with EBER-positive LAHS, the plasma EBV-DNA load is higher. This is primarily because, in non-neoplastic EBV-HLH, the infected cells cannot eliminate EBV, and more EBV remains in the infected cells without leaking into the plasma [23]. Our study further confirmed that the plasma EBV-DNA load was higher in patients with EBER-positive LAHS. Additionally, this study found that platelet count was significantly decreased in patients with EBER-positive LAHS, which may be related to factors such as lymphoma infiltration of the bone marrow, severe cytokine storm suppression of normal hematopoiesis, cytokine-induced destruction of hematopoietic precursor cells, and platelet destruction mediated by overactivated macrophages [24, 25].

Compared with non-neoplastic EBV-HLH, the cytokine levels in EBER-positive LAHS are higher. In our study, the levels of IL-10, IFN-γ, and IL-6 were significantly elevated in the EBER-positive LAHS group, with IL-10 levels notably higher than IL-6. This phenomenon is mainly attributed to the cytokine storm triggered by tumor-related factors and the immune hyperactivation mediated by EBV [26–28]. EBV-infected lymphocytes can secrete large amounts of IFN-γ and IL-10 through the expression of LMP-1 and activation of the NF-kB pathway [29, 30]. At the same time, tumor cells can modulate the immune microenvironment by producing IL-10, causing the immune system to shift towards a suppressed state, thereby promoting immune escape of the tumor [31, 32]. Previous studies have shown that elevated IL-6 and IL-10 levels are independent factors predicting LAHS, with IL-10 levels being three times higher than IL-6, and possessing greater diagnostic value [15]. This conclusion is consistent with the results of our study.

One type of clinical prediction model visualization is the nomogram, which enables doctors to quickly and visually assess whether patients presenting with EBV-associated hemophagocytic lymphohistiocytosis are likely to have EBV-positive lymphomas and to recommend an early course of diagnosis and treatment. Based on platelets < 33.5*10^9^/L, IL-6 > 20.79 pg/ml, IFN-γ > 12.12 pg/ml, and IL-10 > 39.87 pg/ml, a nomogram prediction model was developed in this study to differentiate patients with EBER-positive LAHS from those with non-neoplastic EBV-HLH. In the training set (AUC:0.825, sensitivity:0.778, specificity:0.870) as well as the validation set (AUC:0.812, sensitivity:0.619, specificity:0.875), the model demonstrated strong discrimination. The training and validation sets’ calibration curves demonstrated strong agreement between the model’s predicted and actual probabilities, demonstrating the model’s excellent extrapolation and calibration. The model’s good clinical utility and benefit rate were revealed by the DCA curves.

This study is a single-center retrospective analysis, and due to the relatively low incidence of HLH, the sample size included is limited, which may introduce some bias. Future multi-center prospective studies are needed to externally validate the model and refine its generalizability. Additionally, due to differences in the diagnostic techniques used in various treatment groups at our center, sCD25 and NK cell activity were not included as research variables.

## Conclusions

This study showed that the risk model built using platelets < 33.5*10^9^/L, IL-6 > 20.79 pg/ml, IFN-γ > 12.12 pg/ml, and IL-10 > 39.87 pg/ml could distinguish between EBER-positive LAHS and non-neoplastic EBV-HLH more effectively. It also demonstrated excellent reliability, indicating that the diagnostic prediction model could assist clinicians in early and convenient treatment identification and individualization.

## Data Availability

The datasets used and/or analysed during the current study are available from the corresponding author on reasonable request.

## Abbreviations

ALB: Albumin
ALT: Alanine aminotransferase
APTT: Activated partial thromboplastin time
AST: Aspartate aminotransferase
AUC: Area under the curve
BUN: Blood urea nitrogen
Ca: Calcium
CR: Complete remission
Cr: Creatinine
CRP: C-reactive protein
CXCL: Chemokine ligands
DBIL: Direct bilirubin
DCA: Decision Curve Analysis
EBER: Epstein-Barr virus-encoded RNA
EBV: Epstein-Barr Virus
FDP: Fibrinogen degradation products
FIB: Fibrinogen
HB: Hemoglobin
HLH: Hemophagocytic lymphohistiocytosis
IG: Immunoglobulin
ILs: Interleukins
IM: Infectious mononucleosis
INF-γ: Interferonγ
INR: International normalized ratio
IP-10: Interferon-inducible protein-10
K: Kalium
LAHS: Lymphoma-associated Hemophagocytic Lymphohistiocytosis
LASSO: Least absolute shrinkage and selection operator
LDH: Lactate dehydrogenase
LMP-1: Latent membrane protein-1
Mg: Magnesium
MIG: Monokine induced by interferon gamma
MON: Monocytes
Na: Natrium
NE: Neutrophilicgranulocyte
NF-KB: Nuclear factor kappa B
NR: No reaction
OR: Odds Ratio
PBMC: Peripheral blood mononuclear cell
PCR: Polymerase chain reaction
PET/CT: Positron emission tomography/computedtomography
PLT: Blood platelet
PT: Prothrombin time
ROC: Receiver operating characteristic
PR: Partial remission
SF: Serum ferritin
sHLH: Secondary hemophagocytic lymphohistiocytosis
STB: Serum total bilirubin
TC: Total cholesterol
TG: Triglycerides
TNF-α: Tumor necrosis factor-α
TT: Thrombin time
VP-16: Etoposide
WBC: White blood cell count
